# Effects of a multi-level intervention on pedestrians’ behavior among Iranian health worker supervisors: a randomized controlled trial

**DOI:** 10.3389/fpubh.2025.1485934

**Published:** 2025-05-09

**Authors:** Sepideh Harzand-Jadidi, Maryam Vatandoost, David C. Schwebel, Homayoun Sadeghi-bazargani, Fatemeh Bakhtari Aghdam, Hamid Allahverdipour

**Affiliations:** ^1^Road Traffic Injury Research Center, Tabriz University of Medical Sciences, Tabriz, Iran; ^2^Department of Health Education & Promotion, School of Health, Tabriz University of Medical Sciences, Tabriz, Iran; ^3^Department of Psychology, University of Alabama at Birmingham, Birmingham, AL, United States

**Keywords:** traffic accident, pedestrians, safe behavior, health workers, socio-ecological model, multi-level intervention

## Abstract

**Background:**

Pedestrian injury is a global public health concern. Multi-level community-based health education campaigns offer promise to reduce the burden. The current study investigated the effect of a multi-level intervention based on the socio-ecological model (SEM) to improve pedestrian safety by training health worker supervisors in Iran, with the expectation that they would disseminate their learned knowledge more broadly in the population.

**Methods:**

Eighty-two health worker supervisors in Tabriz, Iran were randomized to an intervention or control group, with 41 assigned to each group. Participants in the intervention group received both live pedestrian safety education and offline training through a virtual WhatsApp group. Messages, pictures, and short videos about pedestrian safety were sent to participants, and solutions were discussed in the group settings. The control group had no training. Self-reported pedestrian behavior was assessed before and after the intervention.

**Results:**

At baseline, there were no significant differences between the intervention (82.48 ± 8.54) and control (81.05 ± 8.55) groups in pedestrian behavior scores (t = 2.01, *p* = 0.001). After the intervention, the average score of pedestrians’ behaviors of the intervention group increased significantly compared to the control group (87.98 ± 5.83 vs. 80.37 ± 9.03), (t = 3.61, *p* = 0.0002). All five subscale scores of PBQ, including adherence to traffic rules and recommendation, violations, positive behaviors, distraction, and aggressive behaviors showed similar and significant changes in the intervention group compared to control group.

**Conclusion:**

Application of a multi-level intervention based on the socio-ecological model improved self-reported pedestrian behavior of health worker supervisors. Using multi-level interventions by targeting health workers, who then disseminate their learning to the public, could enhance pedestrian safety across society.

## Introduction

Motor vehicle crashes (MVCs) are the eighth leading cause of death globally (1). In Iran, the location of the present research, MVCs are the leading cause of years of life lost (YLL) and the second leading cause of death, after cardiovascular disease. Experts estimate that 2.5% of the world’s MVCs occur in Iran (2). Among MVC victims, pedestrians are particularly vulnerable. In fact, over 30% of MVC fatalities in Iran are pedestrians (3, 4). A wide range of factors place pedestrians at risk for MVCs, including distracted behavior while crossing streets, lack of visibility, and pedestrians’ violation of traffic laws (5, 6). Observational data from Iran support the fact that pedestrians take many risks. In one study, almost 60% of the pedestrians committed violations, including crossing in locations without a crosswalk, crossing intersections diagonally, and crossing the street while distracted (7).

Experts suggest several strategies to improve behavior and reduce traffic injuries. One recommendation is implementation and enforcement of legal policies to increase safety in traffic. These have proven effective in many instances, such as a program to increase law enforcement for seat belt use and speed limits along two US highways, which resulted in significant reduction in the number of crashes (8). Another option is to promote safe behaviors through training programs. Zare et al. (9) reported that an educational program was helpful in promoting the street crossing behavior of child pedestrians, for example, and Zhang et al. (10) found that educational campaign for enhancing adult pedestrian safety, improved overall pedestrian behavior, and that the improvement was most significant for places closest to the locations where campaign activities occurred.

A third option is to implement multi-level community-based health education campaigns. These programs are typically based in behavioral theory such as the social-ecological model (SEM), which suggests multi-level change in the individual, social, physical, organizational, and political environments is most likely to successfully influence and change health behaviors (11–20). Such campaigns are frequently used to successfully increase public awareness of healthy behaviors (21, 22) and have proven successful to reduce MVCs (23–25). In the United States, for instance, campaigns were implemented to successfully increase parents’ safety in using child restraint systems (21). Similarly, an educational campaign to raise seat belt use among teenagers was conducted in Colorado for 2 years and resulted in 20% increase in seat belt use (26). Other programs have used community-based educational campaigns to improve motorcycle helmet use in Thailand (27) and Vietnam (28) through strategies like roadside posters and billboards, radio, television, and social media broadcasts.

An effective strategy to accomplish multi-level community-based pedestrian education is through the train-the-trainer model, whereby health workers are trained first, and then they disseminate their training to citizens (29–31). Iranian Ministry of Health staff have potential to achieve multi-level behavior change through their extensive ties to social networks and communities, and therefore the Ministry of Health uses health workers to distribute wide-ranging health-related information to the community (21). Health workers in Iran generally focus on a wide range of physical and mental health topics, but traffic safety is surprisingly omitted. Since health care workers are considered the stewards of health-related education by the national road traffic safety education program, it is necessary to train them concerning traffic safety behaviors (24). This study worked to educate health worker supervisors about safe pedestrian behavior with the objective that they would learn the material and then share it with their teams and in the community via a multi-level interventional program we developed based on SEM, the Iranian Pedestrian Safety Program for Health Workers (IPSP-HW). We hypothesized the IPSP-HW intervention would create pedestrian safety behavior change among the targeted health worker supervisors.

## Materials and methods

### Study design and sampling

**Figure 1:**
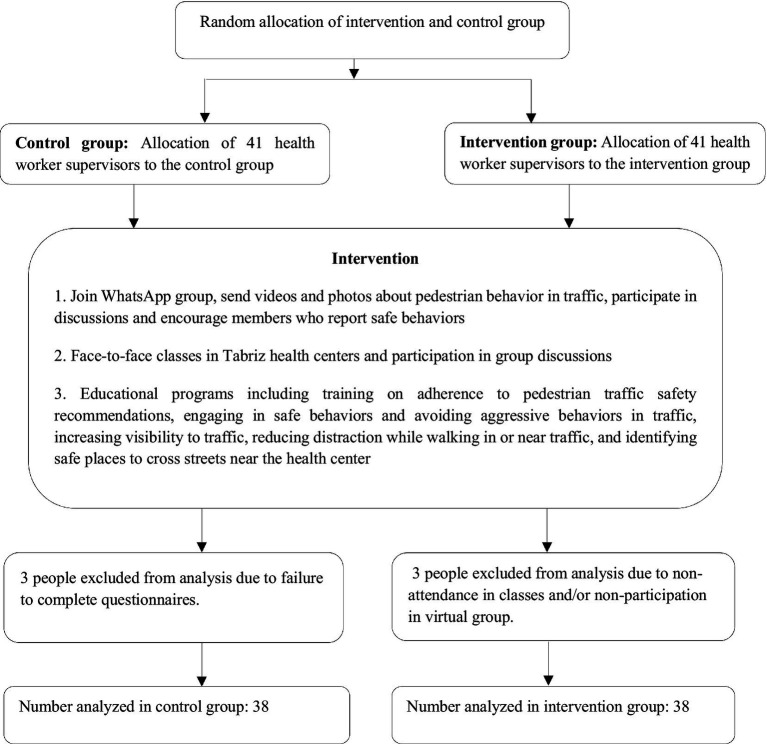
Flowchart of the study.

Health worker supervisors from Tabriz, East Azerbaijan province, Iran, were recruited for a two-stage randomized controlled trial (RCT). At baseline, the health worker supervisors’ pedestrian behavior was assessed through the self-report Iranian Pedestrian Behavior Questionnaire (PBQ; details below). Then, based on the results of this assessment, an intervention program was designed to improve pedestrian behavior among health worker supervisors, with the expectation they would subsequently distribute safety knowledge and encourage behavior change among the populace. Supervisors at 41 health centers in Tabriz were randomly selected as the intervention group and supervisors at a different 41 health centers were randomly selected as the control group. Random assignment was conducted using the website www.random.org with a 1:1 allocation ratio and block size of 6. Random allocation concealment was used. Participant recruitment was straightforward, as all health worker supervisors in Tabriz health centers were eligible. The main challenge was ensuring participant retention, especially in the intervention group, due to the requirement to attend face-to-face sessions and WhatsApp discussions. To address this, reminders were sent via WhatsApp, and participants who missed sessions were directly contacted. Despite these efforts, three participants from each group were excluded due to non-compliance, but this did not affect randomization. Figure 1 displays the study flowchart.

As detailed below, the SEM-based multi-level IPSP-HW intervention involved development of social networks to discuss pedestrian safety through face-to-face classes and a WhatsApp group. Posters regarding safe pedestrian behavior were displayed in the randomly-determined intervention health centers.

Sample size was calculated using the formula of $n={\left(Z_{\alpha /2}+Z_{\beta }\right)}^{2*}\sigma^{2}/{\left(\mu_{1}-\mu_{2}\right)}^{2}$ (32). Assuming a mean change score of 10 in pedestrian safety among the intervention group and no change in the control group, a standard deviation of 21, and a 95% confidence level, a sample size of 34 in each group (68 total) was required to achieve 80% power. Considering there are 82 health centers in Tabriz, the research team enrolled health worker supervisors from each center, yielding a sufficient sample size and allowing for modest attrition. Inclusion criteria included working as the health worker supervisor in a health center, willingness to participate in the study, and the ability to stand and walk without the help of others. Exclusion criteria were minimal: not engaging as a pedestrian in the traffic environment or unwillingness to participate. No participants were excluded for these reasons. We also excluded from analysis any participants in the intervention group who were absent for more than one face-to-face class session or who chose not to participate in virtual discussions and any participants in either group who failed to complete study questionnaires. Three participants were excluded from each group for these reasons. All participants completed informed written consent to participate in the study. The protocol was approved by the Ethics Committee of Tabriz University of Medical Sciences (Reference no. IR.TBZMED.REC.1400.967).

### Instruments

Outcome data were collected both at baseline and following the intervention in both groups through the self-report Iranian Pedestrian Behavior Questionnaire (PBQ), which was developed by Sadeghi-Bazargani et al. (33). Tailored to Iranian culture, the PBQ contains 29 items, each answered on a 5-point Likert scale, and measures self-reported pedestrian behavior in 5 domains: (1) *adherence to traffic safety rules and recommendations* (7 items, e.g., I cross the street after all vehicles are stopped and the pedestrian light is green); (2) *violations* (10 items, e.g., I proceed past signs that say passage forbidden if I feel safe doing so); (3) *distraction* (4 items, e.g., I cross the street while talking on a cell phone or listening to music with my headphones); (4) *positive behavior* (6 items, e.g., I let vehicles pass even if I have the right of way); and (5) *aggressive behavior* (2 items, e.g., If I get angry with a driver’s behavior, I hit or kick their car with my hands or feet). Scores in aggressive behavior, violations, and distraction domains were reversed so that higher scores on all scales reflect safer behaviors. The average content validity coefficient regarding relevancy, clarity, and overall average were 0.86, 0.88, and 0.87, respectively and the questionnaire’s overall Cronbach’s alpha coefficient was 0.84 (33). To calculate the scores presented in Table 1, domain scores were computed by summing the responses for the items within each domain, and the overall pedestrian behavior score was calculated as the sum of these five domain scores. Participants also provided information about their age, level of education, marital status, amount of daily walking, and common methods of transportation.

**Table 1:** Comparison of pedestrian behavior across the two study groups and over time.

Variable		Mean (SD)		Mean Difference (SD)	*t* score over time	p-value*
		Baseline	Post-intervention			
Total score of Pedestrian Behavior	Intervention group	82.48 (8.54)	87.98 (5.83)	5.50 (1.26)	2.01	**0.001**
	Control group	81.05 (8.55)	80.37 (9.03)	0.68 (0.66)	−1.37	0.181
	*t* score between groups	1.13	3.61	2.35	-	-
	p-value**	0.466	**0.0002**	**0.021**	-	-
Adherence to traffic safety rules and recommendations	Intervention	75.03 (12.22)	80 (11.81)	4.97 (2.87)	1.38	**0.017**
	Control	74.88 (10.46)	75.81 (11.81)	−0.93 (0.38)	0.21	0.832
	*t* score between groups	0.24	1.39	0.91	-	-
	p-value**	0.154	**0.016**	**0.036**	-	-
Violations	Intervention	84.52 (11.14)	90.4 (9.05)	5.88 (1.38)	1.77	**0.008**
	Control	82.26 (11.22)	82.14 (11.36)	−0.12 (0.42)	−1.08	0.288
	*t* score between groups	1.42	3.21	1.95	-	-
	p-value**	0.108	**0.002**	**0.005**	-	-
Positive behaviors	Intervention	80.17 (12.61)	83.62 (11.18)	3.45 (2.46)	0.93	**0.036**
	Control	79.12 (10.12)	78.33 (11.98)	0.79 (0.51)	−1.13	0.265
	*t* score between groups	0.05	1.81	1.44	-	-
	p-value**	0.689	**0.007**	**0.053**	-	-
Distraction	Intervention	89.92 (10.05)	93.85 (9.71)	3.93 (2.33)	0.92	**0.034**
	Control	87.08 (11.29)	86.2 (12.22)	0.88 (0.57)	−2.56	0.184
	*t* score between groups	1.59	4.08	2.51	-	-
	p-value**	0.126	**0.001**	**0.044**	-	-
Aggressive behavior	Intervention	94.47 (12.45)	97.85 (7.58)	3.38 (1.41)	−0.41	**0.048**
	Control	94.21 (9.48)	93.57 (10.95)	0.64 (0.65)	−1.29	0.216
	*t* score between groups	0.94	1.40	0.72	-	-
	p-value**	0.917	**0.016**	**0.047**	-	-

*Paired samples *t*-test; **Independent samples *t*-test.

Bold text indicates statistically significant results. Scores for violations and distraction were reversed, such that higher scores indicate safer behavior (less distraction, fewer violations).

### Intervention program (Iranian pedestrian safety program for health workers: IPSP-HW)

Consistent with SEM, the IPSP-HW intervention was designed to incorporate behavior change from the individual, social, and physical environment levels (see Table 2) (34–36). At the individual level, safe behaviors concerning pedestrian crossing were taught in face-to-face classes and via WhatsApp group. At the social level, social networks were created among health worker supervisors in face-to-face classes and the WhatsApp group. Health worker supervisors encouraged each other to practice safe behaviors. At the physical environment level, posters regarding safe places for pedestrian crossing were installed in health centers.

**Table 2:** IPSP-HW Intervention incorporating levels of the socio-ecological model.

Levels of the socio-ecological model	Intervention
Individual level	A virtual group was created on the WhatsApp Text messages, pictures, and short videos were shared in the WhatsApp group. Safe crossing places; safe and positive behaviors in traffic; avoiding aggressive behaviors; rules for pedestrian crossing; the importance of sidewalks; solutions to increase visibility; and the risks of distraction, especially the use of mobile phones while crossing, were discussed. Face-to-face training using a problem-solving approach was conducted in 4 groups in the halls of health centers.
Social environment	Group members rewarded each other with verbal encouragement and informal and emotional support from the manager of the group and their peers as they discussed efforts to increase safe and decrease risky behaviors.
Physical environment	Posters displaying safe pedestrian behaviors were displayed in health centers. SMS texts were delivered to health worker supervisors about safe places for pedestrian-crossing around the health centers. Information about these interventions were posted to the WhatsApp group

Baseline questionnaire data were used to identify the risky pedestrian behaviors health worker supervisors reported engaging in most frequently. Six topics were deemed most critical to address in training: (1) using safe methods of pedestrian crossing, such as avoiding diagonal crossing at intersections and squares and negotiating obstacles like bushes and parked cars; (2) selecting safe places to cross, such as using crosswalks and pedestrian bridges; (3) improving visibility while walking, including use of reflectors and the importance of wearing light-colored clothing; (4) transitioning to safe and positive pedestrian behaviors, such as interacting safely with drivers and other pedestrians and avoiding aggressive behaviors; (5) adherence to traffic safety rules and recommendations, including obeying traffic light signals and checking the environment before entering the street; and (6) avoiding use of distracting mobile phones while walking.

The topics were delivered to health worker supervisors in the intervention group through several means over an 8-week intervention period. First, a WhatsApp group, “Pedestrian Safe Behaviors,” was created. Photos, short videos, and text messages regarding the six target topics were posted by the research team in the WhatsApp group. The members of the WhatsApp group discussed their own safe and unsafe traffic behaviors daily. They shared videos and photos of pedestrians’ behaviors and discussed their efforts to improve safety. As anticipated, the health worker supervisors naturally encouraged each other to practice safe traffic behaviors in the WhatsApp group and congratulated each other for successes, creating a social, emotional, and informational support network that promoted safe pedestrian traffic behavior.

Second, the six topics were taught in an interactive and participatory manner to the health worker supervisors in a face-to-face classroom setting. All training was based on a problem-solving approach (37). Problems relevant to the six pedestrian safety topics were introduced to the health worker supervisors in the form of documentary videos taken by police cameras. Health worker supervisors discussed those problems and identified solutions to them. Following the discussion, researchers shared common causes of pedestrian injuries, prevention strategies, and suitable alternatives to preserve safety.

Third, a poster regarding safe pedestrian behavior was installed weekly at the randomly-assigned intervention group health centers. Posters addressed the 6 target topics and were rotated weekly. Additionally, text messages about safe places to cross streets near the intervention center were sent to the health worker supervisors in the intervention group on two occasions, at the start of the intervention period and again in the middle of it. Two months after the IPSP-HW ended, participants in both groups completed the same self-report questionnaire that they had completed at baseline.

### Data analysis

Data were summarized with mean and standard deviation (quantitative measures) or frequency and percentage (qualitative data). To assure randomization created similar groups, chi-square test was used to compare characteristics of the two groups at the beginning of the study. Primary hypotheses were tested with two series of *t*-tests, paired samples *t*-tests to compare change in outcome measures within the two randomly-assigned groups and independent samples *t*-tests to compare results between the two groups at the end of the study. Stata version 17 was used for all data analyses.

## Results

Table 3 shows demographic characteristics of the participants. As expected through randomization, there were no significant differences between the intervention and control groups. The sample was entirely women, which matches typical demographics of health worker supervisors in Iran.

**Table 3:** Participant characteristics in the intervention and control groups.

Characteristic	Intervention, *n* (%)	Control, *n* (%)	*p*-value^*^
Age (years)			
20–30	7 (18.42%)	1 (2.63%)	0.11
30–40	14 (36.84%)	13 (34.21%)	
40–50	14 (36.84%)	18 (47.37%)	
50–60	3 (7.89%)	6 (15.79%)	
Gender			
Female	38 (100%)	38 (100%)	–
Marital status			
Single	10 (26.32%)	4 (10.53%)	0.07
Married	28 (73.68%)	34 (89.47%)	
Educational level			
Bachelor’s degree	33 (86.84%)	29 (76.32%)	0.23
Master’s degree	5 (13.16%)	9 (23.68%)	
Walking minutes/day			
Less than 30 min	29 (76.32%)	23 (60.53%)	0.13
30 min or more	9 (23.68%)	15 (39.47%)	
Typical transportation			
Personal vehicle	31 (81.58%)	21 (55.26%)	0.06
Public transportation	4 (10.53%)	10 (26.32%)	
Walking	3 (7.89%)	7 (18.42%)	

* Chi-square test was used to compare groups.

Table 1 shows PBQ data for both groups and at both time points, as well as paired-sample t-test results evaluating change over time within groups and independent samples t-test results comparing the two randomly-assigned groups. At baseline, independent samples t-tests confirmed no statistically significant differences between the intervention and control groups in overall pedestrian behavior scores (82.48 ± 8.54 vs. 81.05 ± 8.55, t = 1.13, *p* = 0.466) or in any of the five subscales (*p*-values ranging from 0.108 to 0.917). This indicates that randomization successfully created comparable groups before the intervention.

Following the intervention, paired sample t-tests indicate change over time in the intervention group but not in the control group. As shown, in the intervention group both overall pedestrian behavior (from 82.48 ± 8.54 to 87.98 ± 5.83, t = 2.01, *p* = 0.001) and all 5 PBQ subscales (*p* values range from 0.008 to 0.048) showed significant change from baseline to post-intervention. There were no significant changes in the control group for the overall pedestrian behavior score (from 81.05 ± 8.55 to 80.37 ± 9.03, t = −1.37, *p* = 0.181) or any of the 5 subscales (*p* values range from 0.184 to 0.832).

**Figure 2:**
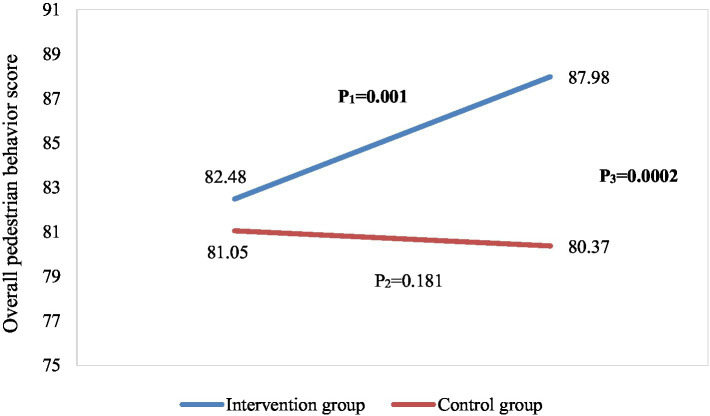
Changes of overall pedestrian behavior across the two study groups and over time. P_1_: *p*-value of paired samples *t*-tests in the intervention group, P_2_: *p*-value of paired samples *t*-tests in the control group, P_3:_ *p*-value of independent samples *t*-tests at the post-intervention stage.

Independent samples t-test results comparing the two randomly-assigned groups were statistically significant at all post-intervention assessment points, both for the overall pedestrian safety score (87.98 ± 5.83 in IPSP-HW intervention group vs. 80.37 ± 9.03 in control group, t = 3.61, *p* = 0.0002) and for all 5 PBQ subscales (*p* values range from 0.001 to 0.016). As expected, the comparison between randomly-assigned groups was not significant at baseline for the overall pedestrian behavior score (82.48 ± 8.54 in IPSP-HW vs. 81.95 ± 8.55 in control, t = 1.13, *p* = 0.466) or for any of the 5 subscales (p values range from 0.126 to 0.917). Figure 2 displays changes in the total pedestrian behavior score graphically.

## Discussion

Our results suggest the SEM-based IPSP-HW intervention was effective in improving pedestrian safety behaviors among Iranian health worker supervisors. The average pedestrian behavior scores were similar across randomly-assigned groups at baseline (*p* = 0.466) and statistically different after the intervention (*p* = 0.0002), with scores in the IPSP-HW intervention group increasing an average of over 5 points (*p* = 0.001). The most substantial improvements were observed in the “adherence to traffic safety rules and recommendations” and “violations” subscales. These two domains showed the highest increases in mean scores post-intervention, indicating that participants in the intervention group exhibited significantly safer behaviors in following traffic rules and reducing pedestrian violations. Other subscales, including “distraction,” “positive behavior,” and “aggressive behavior,” also showed statistically significant improvements, but the effect sizes were comparatively smaller.

Our findings replicate previous research suggesting multi-level education is effective to alter health risk behavior among a target group (38, 39). At the individual level, we were successful in training health worker supervisors to refrain from crossing streets distracted and adhere to traffic regulations. At the physical environmental level, we successfully introduced information concerning safe locations for pedestrians to cross the street. At the social environment level, we were successful in training health worker supervisors to engage in positive pedestrian behaviors such as interacting safely with drivers and avoiding aggressive behavior. The various levels of a multi-level interventions have synergistic effects and can be jointly effective in enhancing health behaviors (5, 11, 12, 14, 18–20).

The Ministry of Health is considered the primary steward of traffic safety behavior training in Iran’s national road traffic safety education program (24), and health workers offer a unique strategy to train the populace in pedestrian safety behaviors (40). In Iran, it is common to teach people health-related behaviors through health workers and the train-the-trainer model. The model has been demonstrated effective in a range of settings and on a range of topics (29–31). MVCs are the second leading cause of death in Iran, but health care workers do not typically offer training in safe traffic behaviors. The results of the present study suggest the training of health worker supervisors has potential to affect their traffic behavior. With broad training, health worker supervisors could train the employees working under them in public health worker offices, who could then spread training for safe traffic behaviors among the public (29–31). Repeated research suggests people change their behavior to match the behavior of others. Through initiation of safe behavior by some individuals, especially respected individuals like health workers, society-wide behavior change can be created (5, 11, 12, 14, 16–19).

The WhatsApp-based virtual training in the intervention proved effective due to several key factors. It offered flexibility, allowing participants to engage with content at their own pace, which was essential for busy health worker supervisors. The platform fostered social support, where participants shared experiences, creating a sense of accountability and motivation. Continuous engagement was maintained through regular updates and peer discussions, which reinforced learning. Additionally, participants shared positive examples of safe behaviors, promoting peer modeling. The cost-effectiveness and scalability of WhatsApp-based training enabled widespread reach with minimal logistical effort, contributing to the intervention’s success. Given its effectiveness, we can consider this platform in future projects as a scalable and cost-efficient tool for health behavior interventions. Scaling up the WhatsApp-based virtual training may face barriers such as limited access to technology in some regions, cultural and linguistic differences, health literacy challenges, and resistance to technology, especially among older adults. Resource constraints, insufficient training capacity, and the need for continuous engagement also pose challenges. Additionally, adapting the intervention to local contexts, including traffic regulations and safety issues, will be essential for its effectiveness.

The current study offers several strengths. It introduced a comprehensive, theory-based multi-level intervention to alter the traffic behavior of health worker supervisors and intervened to promote safe traffic behaviors among them. The training was problem-based and both individualized and socially interactive. The study also had limitations. First, we relied entirely on self-report questionnaires to assess outcomes. Future research should consider behavioral or observational measures of pedestrian behavior to assess intervention effectiveness. Second, our sample was entirely women. Although this is consistent with the composition of health workers in Iran, future research may consider ways to incorporate training of men into the program.

## Conclusion

The present study improved the self-reported pedestrian behavior of health worker supervisors in Iran using a theory-based intervention, IPSP-HW that incorporated training of safe pedestrian behaviors, creating and developing social networks, leveraging virtual networks to increase social support for safe behaviors, and identifying safe locations for pedestrian crossing. Utilization of multi-level interventions by targeting health workers, who then disseminate their learning to the public through a train-the-trainers model, could improve pedestrian safety across society.

## Data Availability

The original contributions presented in the study are included in the article/supplementary material, further inquiries can be directed to the corresponding author.
